# Effectiveness of an antimicrobial treatment scheme in a confined glanders outbreak

**DOI:** 10.1186/1746-6148-8-214

**Published:** 2012-11-07

**Authors:** Muhammad Saqib, Ghulam Muhammad, Abeera Naureen, Muhammad H Hussain, M Nadeem Asi, M Khalid Mansoor, Mehdi Toufeer, Iahtasham Khan, Heinrich Neubauer, Lisa D Sprague

**Affiliations:** 1 Department of Clinical Medicine and Surgery, University of Agriculture, Faisalabad 38040, Pakistan; 2 Department of Clinical Medicine, University of Veterinary and Animal Science, Lahore, Pakistan; 3 Institute of Microbiology, Faculty of Veterinary Science, University of Agriculture, Faisalabad 38040, Pakistan; 4 Friedrich-Loeffler-Institut, Federal Research Institute for Animal Health, Institute for Bacterial Infections and Zoonoses, Naumburger Str. 96a, D-07743 Jena, Germany; 5 Department of Epidemiology and Public Health, University of Veterinary and Animal Science, Lahore, Pakistan

**Keywords:** *Burkholderia mallei*, Zoonosis, Glanders, Horse, Treatment

## Abstract

**Background:**

Glanders is a contagious and fatal zoonotic disease of solipeds caused by the Gram-negative bacterium *Burkholderia (B.) mallei*. Although regulations call for culling of diseased animals, certain situations e.g. wild life conservation, highly valuable breeding stock, could benefit from effective treatment schemes and post-exposure prophylaxis.

**Results:**

Twenty three culture positive glanderous horses were successfully treated during a confined outbreak by applying a treatment protocol of 12 weeks duration based on the parenteral administration of enrofloxacin and trimethoprim plus sulfadiazine, followed by the oral administration of doxycycline. Induction of immunosupression in six randomly chosen horses after completion of treatment did not lead to recrudescence of disease.

**Conclusion:**

This study demonstrates that long term treatment of glanderous horses with a combination of various antibiotics seems to eliminate the agent from the organism. However, more studies are needed to test the effectiveness of this treatment regime on *B. mallei* strains from different endemic regions. Due to its cost and duration, this treatment can only be an option in certain situations and should not replace the current “testing and culling” policy, in conjunction with adequate compensation to prevent spreading of disease.

## Background

Glanders is a contagious and highly fatal zoonotic disease of solipeds caused by the Gram-negative bacillus *Burkholderia* (*B*.) *mallei*. This disease is characterised by progressive loss of efficiency due to the development of nodular and ulcerative lesions in the skin and the upper respiratory tract. The horse is the only known natural host of *B. mallei*. In humans, glanders is usually an occupational disease, affecting individuals in close contact with infected animals such as farmers, grooms, and veterinarians, but also laboratory personnel handling the agent. Glanders has a 95% case fatality rate in untreated septicaemic infections and a 50% case fatality rate in treated human individuals, even if diagnosed early on [1,2]. Despite several studies demonstrating the susceptibility of *B. mallei* to numerous antibiotics *in vitro*, many failed to eradicate the agent from the organism of laboratory animals when challenged [1,3,4]. Current treatment protocols for human glanders therefore, are based on data gained from treatment schemes for melioidosis, a disease caused by the closely related agent *B. pseudomallei*, using a combination of antibiotics for an extended period of time.

In Pakistan, legislation based on the “Glanders and Farcy Act of 1899” calls for the detection and destruction of glanderous animals [5]. However, although animal owners are paid compensation for having their animals culled, the sum is so ridiculously low (~50 PKR; ~0.42 €; ~0.53 US$) that owners prefer to sell their animals beforehand and thereby risk the introduction of disease to hitherto glanders-free regions [6,7]. In view of the difficulties involved in the implementation of regulations in many countries, it thus seems reasonable to re-evaluate the role of [modern] chemotherapy as a possible alternative to culling. Effective treatment schemes and post-exposure prophylaxis are urgently required especially in equestrian sport and breeding where highly valuable animals from endemic regions travel to non endemic countries and vice versa. Finally, although public health must be paramount, greater emphasis has to be placed upon animal welfare and new guidelines developed and implemented in the treatment and prevention of animal disease [8].

The present report describes a treatment protocol of 12 weeks duration based on the parenteral administration of enrofloxacin and trimethoprim + sulfadiazine, followed by the oral administration of doxycycline during a confined glanders outbreak in horses in Lahore, Pakistan.

## Animals, materials and methods

### Study animals and study area

Twenty three horses (15 male, 8 female; median age 3.5 year; median body weight 340 kg) were found to have contracted glanders during an outbreak at the Lahore Polo Club (LPC) in Lahore, Pakistan in 2005 (Table 1). Questioning of the staff revealed that all affected horses had drunk water from a common water trough. After cultural and diagnostic confirmation of glanders by means of an intradermo-palpebral mallein test [9-11], the horses were transferred to the Department of Clinical Medicine and Surgery (CMS), University of Agriculture (UAF), Faisalabad, Pakistan for experimental therapy. During the treatment period, the horses were managed at the Large Animal Indoor Facility of the Department. The facility was declared off-limits for all personnel other than the investigators and attendants of the equines; relevant bio-security measures were initiated.

**Table 1:** Description of the polo horses included in the study

**#**	**Sex**	**Age (years)**	**Weight [~ kg]**	**Breed**	**CSS**	**BCS**	**Onset of disease (weeks)**	**Isolation *B. mallei *from**	**Necropsy findings**	**Current where abouts**
1	f	6	367	English	1	4	1	NS, CN		LPC^§^
2	g	7	371	Thorough	2	3	1	NS, CN		LFU
3*	g	6.5	316	ND	3	2	2	NS, CN;VB	PTC	-
4	f	5	360	Thorough	1	4	1.5	CN		LPC
5	m	8	347	Anglo-Arab	2	2	2	NS, CN		LFU
6*	f	5.5	371	Thorough	2	3	2.5	NS, CN		
7*	g	6	328	Arab	3	2	1	NS, CN	PTC	-
8*	f	5	353	Thorough	1	4	2	NS	PTC	-
9	g	3.5	339	Arab	2	3	2	NS, CN		LFU
10	m	7	370	ND	2	3	2	NS, CN		LFU
11	g	5	332	Arab	3	2	2	NS, VB		LPC
12	g	5	340	Arab	1	3	2	NS		LFU
13	m	8.5	365	Thorough	2	3	1.5	NS		LFU
14	g	7.5	340	Thorough	3	2	2	NS, VB		LPC
15	m	7	359	Thorough	2	4	1	NS		LFU
16^#^	f	5	370	Thorough	1	4	1	NS		LFU
17	g	7	319	Anglo-Arab	3	2	3	NS, CN		LPC
18	f	5.5	373	Anglo-Arab	2	4	1	NS		LPC
19	f	4.5	310	Arab	3	2	2	NS, CN		LFU
20*	g	5	368	Arab	1	4	5 d	NS		-
21	g	6	348	Anglo-Arab	2	3	1.5	NS, CN		LPC
22*	f	6	329	Arab	4	2	3.5	NS, CN, VB	PTC	-
23	g	7	370	Thorough	1	5	1	NS		LFU

*M* male, *g* gelding, *f* female, * euthanized horses, *#* aborted during treatment, *NS* nasal discharge swab, *CN* cutaneous nodule aspirate, *VB* venous blood sample, *PTC* pulmonary tissue calcification, *LPC* Lahore Polo Club, *LFU* Lost during follow-up, *§* 2 foals since 2007.

### Animal ethical issues

The present study was approved by the Synopsis Scrutiny Committee and the Animal Ethics Committee, Faculty of Veterinary Science, UAF, Faisalabad, Pakistan. Permission for the experimental treatment was additionally granted by the Secretary of Livestock and Dairy Development, Government of Punjab, Pakistan.

### Bacteriological cultivation and identification of B. mallei

Culture and identification of *B. mallei* was performed as described previously [11] Briefly, nasal swabs, nodule aspirates and venous blood samples collected from the horses were plated onto brain heart infusion agar (BHI; Oxoid Limited, Basingstoke, UK) supplemented with 5% defibrinated sheep blood, BHI-4% glycerol and MacConkey agar (Oxoid Limited). In order to minimise contamination, each nasal swab was incubated at room temperature in distilled water containing 3000 IU/mL benzyl penicillin for 3 h prior to plating. Culture plates were incubated at 37°C under aerobic conditions for 72 h. Representative colonies of *B. mallei* were screened for Gram staining, catalase, indole tests, and colistin resistance. The organism was presumptively identified as *B. mallei* if bipolar, catalase positive, indole negative, colistin resistant and if an irregularly, Gram-negative stained rod. Isolates were then subjected to motility testing, triple sugar iron slants (Oxoid Limited) and API 20E (Bio Mérieux, Craponne, France). Non motile, triple sugar iron negative, arginine, and gelatine positive isolates were finally identified as *B. mallei*.

### Susceptibility testing, antibiotics, dose, and treatment regime

Antibiotics were selected on the basis of susceptibility testing by means of the disc diffusion method [12]. In brief, MIC values of 41 *B. mallei* isolates from various Glanders outbreaks in the Pakistani Punjab including those from the present outbreak were determined by agar dilution for 20 different antimicrobials: ampicillin, amoxicillin, co-amoxiclav, ceftiofur, doxycycline, oxytetracycline, cephradine, ceftriaxone, ciprofloxacin, sulphadiazine (SDZ), trimethoprim, roxithromycin, clarithromycin, cefotaxime, cefuroxime, ciprofloxacin, enrofloxacin, norfloxacin, and chloramphenicol. The isolates were susceptible to amoxicillin-clavolanic acid, doxycycline, chloramphenicol, gentamycin, and trimethoprim-sulphadiazine. The isolates obtained from the horses of the present study were additionally resistant to enrofloxacin. Mode MICs for these antimicrobials were 2, 1, 8, 4, 1, and 2 mg/mL, respectively [11].

A twelve week course of antibiotics was applied. In week 1, horses were treated intravenously with 8 mg/kg body weight enrofloxacin (Enrotil 10%, Dae Sung Microbiological Labs, Korea) and 32 mg/kg body weight Tribrissen® 48% (trimethoprim (TMP) + sulphadiazine; Glaxo-Smith-Kline, Pakistan) once a day. In weeks 2 and 3, the dosage was halved to 4 mg/kg body weight enrofloxacin and 16 mg/kg body weight Tribrissen® 48%. During weeks 4–12, the horses were given 6 mg/kg body weight doxycycline orally (Doxy-veto, VMD, Arendonk, Belgium) twice a day.

### Recording of clinical signs

Clinical signs such as fever, inappentence, debility, cough, dyspnoea, nasal discharge, development and location of ulcers, adspection of lymph nodes and lymphatics were recorded prior to (d 0), during and after treatment (Table 2).

**Table 2:** Clinical signs observed in polo horses with glanders during the study period

	**Clinical signs**	**d 0 before malleinisation**		**d 60 during treatment**		**d 180 post-treatment**		**d 360 follow-up period**	
		**#**	**%**	**#**	**%**	**#**	**%**	**#**	**%**
a)	Fever	22	95.6	-		-	-	-	-
	mild (38.9-39.4°C; 102.0 -102.9°F)								
	moderate (39.5-40.0°C; 103.1-104.0°F)								
b)	Inappentence	23	100	-	-	-	-	-	-
c)	Debility	23	100	-	-	-	-	-	-
d)	Quick loss of stamina (when put to work, exercised or driven)	23	100	-	-	-	-	-	-
f)	Cough	14	60.9	-	-	-	-	-	-
g)	Dyspnoea	16	69.6	-	-	-	-	-	-
h)	Abnormal respiratory sounds (audible at a distance)	10	43.5	-	-	-	-	-	-
i)	Nasal discharge	18	78.3	-	-	-	-	-	-
	- Uni-lateral	10	43.5	-	-	-	-	-	-
	- Bilateral	8	34.8	-	-	-	-	-	-
	- Without blood	4	17.4	-	-	-	-	-	-
	- With blood	14	60.9	-	-	-	-	-	-
	- Occasional epistaxis (when put to work, exercised or driven)	6	26.1	-	-	-	-	-	-
j)	Nasal septum ulcers	16	69.6	-	-	-	-	-	-
k)	Nodules and crater-like ulcers	20	86.9	-	-	-	-	-	-
	- Medial aspect of thigh	14	60.9	-	-	-	-	-	-
	- Perianal region	6	26.1	-	-	-	-	-	-
	- Groin	6	26.1	-	-	-	-	-	-
	- Below hock	6	26.1	-	-	-	-	-	-
	- Foreleg	2	8.7	-	-	-	-	-	-
	- Achilles tendon	2	8.7	-	-	-	-	-	-
	- Face	4	17.4	-	-	-	-	-	-
	- Testes	4	17.4	-	-	-	-	-	-
	- Distributed almost all over the body	4	17.4	-	-	-	-	-	-
l)	Orchitis	4	26.6	-	-	-	-	-	-
m)	Enlargement of submaxillary lymph nodes	23	100	-	-	-	-	-	-
	- Enlargement visible from a distance	4	17.4	-*	-	-	-	-	-
	- Enlargement detectable only on palpation	20	86.9	-*	-	-	-	-	-
n)	Cording of lymphatics	10	43.5	-	-	-	-	-	-
	- Inner aspects of thigh	6	26.1	-	-	-	-	-	-
	- Latero-medial aspects of trunk and abdomen	4	17.4	-	-	-	-	-	-
o)	Oedema of hind legs	8	34.8						

#: number of horses affected; *: tortuous, palpably enlarged; - not observed.

### Assessment of treatment response

Response to treatment was evaluated by means of daily monitoring and recording of rectal temperature, pulse, and respiration rates during parenteral therapy. Changes in nutritional status were calculated by means of body condition scores (BCS) for equines according to Henneke et al., [13]. These scores were then juxtaposed to the clinical severity score (CSS) associated with the health status at day 0 (i.e. prior to treatment) and at intervals of 60 days during and up to 1 year after treatment. Blood analyses were carried out at day 0 prior to treatment for base line values, at completion of treatment (d 84) and then at 90 day intervals during the one year follow-up period according to the procedures previously described [14]. Indirect haemagglutination assays (IHA) using *B. mallei* strains PRL4, PRL7 and China 5 [11] were carried out on d 0 (prior to malleinisation), during treatment and then at 30 day intervals throughout the follow-up period (Table 3).

**Table 3:** IHAT antibody titres of polo horses with glanders during the study period

**Treatment period (months) n = 23**					**Post treatment observation period (months) n = 17**											
**titer**	**d0**^**#**^	**1**	**2**	**3 (d 84)**	**1**	**2**	**3**	**4**	**5**	**6**	7*	**8**	**9**	**10**	**11**	**12**
**1:40**	-	-	-	-	-	-	-	-	-	-	-	-	-	-	-	-
**1:80**	-	-	-	-	-	-	-	-	-	-	-	2	2	6	7	8
**1:160**	1	-	-	-	-	-	-	-	1	-	5	12	15	11	10	9
**1:320**	-	-	-	-	-	-	6	14	11	15	10	3	-	-	-	-
**1:640**	7	-	-	8	1	7	11	3	5	2	-	-	-	-	-	-
**1:1280**	10	6	12	8	10	9	1	-	-	-	-	-	-	-	-	-
**1:2560**	4	10	11	7	6	-	-	-	-	-	-	-	-	-	-	-
**1:5120**	-	4	-	-	-	-	-	-	-	-	-	-	-	-	-	-
**1:10240**	1	3	-	-	-	-	-	-	-	-	-	-	-	-	-	-
**1:20480**	-	-	-	-	-	-	-	-	-	-	-	-	-	-	-	-

^#^prior to malleinisation; *values missing due to loss of samples.

### Assessment of absence of disease

Horses were observed for recrudescence of disease during the one year follow-up period. The study animals were subjected to a mallein test at completion of treatment (day 84) and at the end of the one year follow-up period. Additionally, on day 90, six horses were immunocompromised by daily intramuscular administration of 14 mL Penacort (2.5 mg Dexamethasone + 7.5 mg Prednisolone/mL; Selmore Pharmaceuticals, Pakistan) for 10 days. Horses were exercised twice daily for one hour. On days 100, 102 and 104, two horses each were euthanized by the i. v. administration of sodium pentothal (3 g; Thiopental, Rotexmedica, Trittau, Germany) and magnesium sulphate (200 g; Shamsi Pharmacy, Lahore, Pakistan). In order to exclude the possibility of carrier status among the treated horse, 110 clinically healthy, glanders negative, i.e. mallein test negative sentinel horses were housed with the treated horses during the one year follow-up period.

### Bacterial cultivation upon necropsy

From the six euthanized horses, tissue samples were taken aseptically from the mediastinal and submandibular lymph nodes, as well as from the lung. Bacterial cultivation was carried out as previously described [9-11]. Briefly, ~7 g tissue slices were placed in 5 mL PBS and homogenised using a Pyrex Potter-Elvehjem tissue homogeniser with a PFTE pestle (17 mL capacity; Corning, Lowell, MA, USA). From this homogenate 5 mL triplicates were subsequently cultured in 100 mL BHI plus 2% glycerol (UH0, Oxoid Limited), containing 100 U/mL penicillin G, and 10 U/mL colistin sulphate (both Sigma Aldrich, Taufkirchen, Germany) for 56 h at 37°C. Next, 200 μL from this broth were inoculated in duplicate on BHI agar supplemented with 5% defibrinated sheep blood and incubated for 6 days at 37°C.

## Results

### Isolation of B. mallei from clinical samples

*B. mallei* was isolated from 22/23 nasal swabs, 13/23 aspirates from cutaneous nodules and 4/23 venous blood samples (Table 1).

### Clinical signs observed in the horses throughout the study period

Clinical signs in the diseased horses were recorded at the time of diagnosis (d 0), during treatment (parenteral & oral), at 6 months after treatment and during the 1 year follow-up observation period (Table 2). Prior to treatment fever was observed in 22/23 horses, inappentence in 23/23, debility in 23/23, loss of stamina in 23/23, cough in 14/23, dyspnoea in 16/23, nasal discharge in 18/23, nasal septum ulcers in 16/23, nodules and ulcers on the body in 20/23, orchitis in 4/23, enlargement of submaxillary lymph nodes in 23/23 and cording of lymphatics of hind limbs and ventro-lateral abdomen in 10/23 horses.

After the first week of treatment, the clinical picture improved visibly. Fever subsided and only slightly increased rectal temperature was observed in 9/23 horses (Table 4). Horses began to regain their appetite, respiration rates started improving with 10/23 showing physiological values (Table 5). By the end of treatment week one, nasal septum ulcers had healed in 16/16 horses and the oedema in the hind legs were only slightly visible in the eight affected horses. Nodules and ulcers were cleared in 19/20 horses and by the end of treatment week three in all horses (Table 2). Pulse rates returned to physiological values by the end of treatment week two in 22/23 horses (Table 6). With completion of treatment week three, moderately enlarged submaxillary lymph nodes were still palpable in 19/23 horses; all animals showed regular food intake. After treatment week 12, no clinical signs were observed in any of the horses; however, enlarged lymph nodes were still palpable in 9 horses (Table 4).

**Table 4:** Rectal temperatures (°F) during parenteral treatment with enrofloxacin and Tribrissen® 48%

	**Rectal temperature (°F)**																					
	**Week 1 (d)**								**Week 2 (d)**							**Week 3 (d)**						
**Horse**	**0**	**1**	**2**	**3**	**4**	**5**	**6**	**7**	**8**	**9**	**10**	**11**	**12**	**13**	**14**	**15**	**16**	**17**	**18**	**19**	**20**	**21**
**1**	104	103.8	103	103.2	102.4	101.2	100.8	**100**	**100.4**	**Normal body temperature**												
**2**	103.2	103.8	102.2	102.8	101.2	102	101.6	100.8	**100**	**Normal body temperature**												
**3**	103.4	101.4	101.8	102.6	101	100.8	100.8	**100**	**Normal body temperature**													
**4**	100.8	102.4	101	101.8	**99.8**	**100.2**	**99.8**	**Normal body temperature**														
**5**	102.8	101.6	101.6	101.2	100.2	100.8	**100**	**Normal body temperature**														
**6**	101.8	102.4	102	102.6	101.8	101.4	101.2	100.6	**99.8**	**Normal body temperature**												
**7**	103	103.2	103	102.8	102	101.8	101.8	101	101.2	**100.8**	**100**	**Normal body temperature**										
**8**	102.4	101.4	101.8	101	101.4	100.2	**100.4**	**100**	**Normal body temperature**													
**9**	103.4	103	102.8	101.6	**100.2**	**100.8**	**100**	**Normal body temperature**														
**10**	105	103.4	104.2	103	103.6	102.8	101.6	100.8	101	**100**	**100.8**	**Normal body temperature**										
**11**	103.8	101.6	102	99.8	**100.2**	**99.2**	**100**	**Normal body temperature**														
**12**	102.8	102.6	102.8	101.2	101.6	100.2	**100.6**	**100.2**	**Normal body temperature**													
**13**	104.2	104	104.2	104	103.2	102.6	102	**100**	**99.8**	**Normal body temperature**												
**14**	102.4	101.8	102	101	**100**	**100.8**	**100.2**	**Normal body temperature**														
**15**	105.6	103.8	102.8	101.6	102	101	100.2	**100.6**	**100.6**	**Normal body temperature**												
**16**	102.2	101.8	101	101	**99.8**	**100.6**	**99**	**Normal body temperature**														
**17**	106	104.2	103.2	103.8	102.8	103	101	101.8	101.4	101	**100.2**	**100.6**	**Normal body temperature**									
**18**	103.4	103	103.2	102	101.2	101	100.8	**100**	**Normal body temperature**													
**19**	104	104.6	103.8	104	102.6	102	102.6	100	**99**	**98.9**	**Normal body temperature**											
**20**	103.6	101	102.2	101	102	102.2	100.2	100.8	**100.6**	**Normal body temperature**												
**21**	105.4	103.8	104.8	102.6	103.8	102	101.6	102.2	**100**	**100**	**Normal body temperature**											
**22**	103.6	101.8	102.4	102.4	102	101.8	102	101.8	101.6	**99**	**100.2**	**Normal body temperature**										
**23**	104.6	103.8	102.8	102.6	101.2	100.2	100.8	101	**100.4**	**100**	**Normal body temperature**											

*Physiological rectal temperature (°F) in an adult horse: 99–100.5 [15].

**Table 5:** Respiration rates during parenteral treatment with enrofloxacin and Tribrissen® 48%

	**Respiration rate (per minute)**																					
	**Week 1 (d)**								**Week 2 (d)**							**Week 3 (d)**						
**Horse**	**0**	**1**	**2**	**3**	**4**	**5**	**6**	**7**	**8**	**9**	**10**	**11**	**12**	**13**	**14**	**15**	**16**	**17**	**18**	**19**	**20**	**21**
**1**	20	21	19	18	20	18	18	17	18	**16**	**14**	16	18	18	**15**	**12**	**Normal respiration rate**					
**2**	24	19	21	20	19	16	18	**12**	**15**	**13**	**Normal respiration rate**											
**3**	48	40	46	36	29	24	26	30	22	23	20	**15**	18	16	16	**13**	16	**Normal respiration rate**				
**4**	20	20	18	16	16	**11**	15	**Normal respiration rate**														
**5**	33	31	34	32	30	21	20	20	20	21	20	17	**13**	16	16	**14**	16	**11**	**14**	**Normal respiration rate**		
**6**	28	21	23	21	17	**15**	15	10	**Normal respiration rate**													
**7**	28	32	29	24	24	20	20	21	24	24	20	16	19	**15**	16	**13**	**15**	**Normal respiration rate**				
**8**	48	32	24	24	24	19	**15**	16	**13**	**13**	**Normal respiration rate**											
**9**	32	32	32	27	28	20	20	21	20	17	18	18	**15**	**14**	**Normal respiration rate**							
**10**	22	20	16	20	16	**15**	18	18	14	**Normal respiration rate**												
**11**	24	24	25	24	20	20	16	**13**	**15**	16	13	**Normal respiration rate**										
**12**	29	32	30	21	28	23	22	21	23	19	21	22	20	20	17	19	18	**15**	17	**11**	**13**	**Normal**
**13**	28	29	26	28	27	22	17	20	16	16	**15**	17	19	18	**12**	**15**	**Normal respiration rate**					
**14**	28	22	22	21	20	18	21	21	18	19	20	20	**15**	**13**	12	**Normal respiration rate**						
**15**	35	36	30	32	28	24	20	19	20	16	17	17	**15**	**13**	**Normal respiration rate**							
**16**	28	21	18	19	**13**	**15**	**Normal respiration rate**															
**17**	20	20	19	20	17	**13**	15	**13**	**14**	**Normal respiration rate**												
**18**	23	21	21	24	20	18	17	18	18	**15**	18	20	**15**	13	**Normal respiration rate**							
**19**	20	22	20	21	20	20	22	21	18	20	16	16	18	**14**	**15**	**Normal respiration rate**						
**20**	18	18	16	17	16	18	**15**	17	**13**	**15**	**Normal respiration rate**											
**21**	24	24	24	23	21	20	19	20	18	**13**	**15**	12	12	**Normal respiration rate**								
**22**	28	26	25	22	22	19	20	17	16	**15**	**15**	15	**Normal respiration rate**									
**23**	24	20	20	21	20	16	18	16	**15**	17	**13**	**12**	15	**Normal respiration rate**								

*Physiological respiration rate (breath/min) in an adult horse: 10–15 [15].

**Table 6:** Pulse rate (beat/min) during parenteral treatment with enrofloxacin and Tribrissen® 48%

	Pulse rate* (per minute)																					
	**Week 1 (d)**								**Week 2 (d)**							**Week 3 (d)**						
**Horse**	**0**	**1**	**2**	**3**	**4**	**5**	**6**	**7**	**8**	**9**	**10**	**11**	**12**	**13**	**14**	**15**	**16**	**17**	**18**	**19**	**20**	**21**
**1**	48	52	44	40	36	44	40	32	40	36	36	**Normal pulse rate**										
**2**	68	76	60	60	56	60	44	48	40	40	40	44	32	28	**Normal pulse rate**							
**3**	80	72	64	72	72	56	60	52	56	48	48	40	36	44	36	40	36	32	40	**Normal pulse rate**		
**4**	52	48	48	52	44	40	44	40	44	40	36	40	**Normal pulse rate**									
**5**	64	68	60	60	48	52	52	52	48	44	44	36	40	**Normal pulse rate**								
**6**	60	68	60	56	60	44	40	48	40	40	**Normal pulse rate**											
**7**	64	64	64	60	60	52	48	52	56	52	44	44	36	40	44	40	36	**Normal pulse rate**				
**8**	48	40	44	48	40	40	44	40	44	48	44	40	40	**Normal pulse rate**								
**9**	88	100	96	96	88	96	64	64	72	70	58	44	44	48	40	44	40	**Normal pulse rate**				
**10**	40	60	72	64	68	52	56	52	44	52	48	36	44	40	32	36	**Normal pulse rate**					
**11**	68	60	72	68	52	48	44	56	40	44	40	40	**Normal pulse rate**									
**12**	44	40	44	40	52	40	44	36	48	48	40	44	36	**Normal pulse rate**								
**13**	64	64	68	56	56	44	52	48	40	48	44	44	44	36	36	32	**Normal pulse rate**					
**14**	60	40	48	36	40	40	44	36	40	**Normal pulse rate**												
**15**	100	72	96	100	82	72	68	64	56	60	52	44	44	52	40	40	**Normal pulse rate**					
**16**	60	48	48	40	36	48	36	40	**Normal pulse rate**													
**17**	64	72	76	72	52	52	40	52	48	44	40	48	48	40	36	44	40	44	48	44	44	**Normal pulse rate**
**18**	36	48	40	36	32	36	32	**Normal pulse rate**														
**19**	60	72	60	80	72	48	52	52	56	60	56	52	48	44	40	44	48	40	44	40	**Normal pulse rate**	
**20**	72	48	56	52	52	44	48	40	40	**Normal pulse rate**												
**21**	80	72	64	56	60	52	52	48	48	40	44	44	52	48	40	32	**Normal pulse rate**					
**22**	48	44	44	44	48	40	44	48	40	44	40	36	**Normal pulse rate**									
**23**	56	52	48	48	52	44	36	28	40	40	36	**Normal pulse rate**										

*Physiological pulse rate (beat/min) in an adult horse: 30–40 [15].

Of note is that on treatment day 11 one horse became restless and showed signs of muscular fasciculation in the forelimbs during the application of Tribrissen® 48%. The injection was stopped immediately. The adverse reaction subsided within three hours and the horse was re-injected three hours later. All horses developed alopecia on the trunk and neck during the second week of treatment. Hair growth resumed on week 2 after termination of intravenous therapy. One mare, whose pregnancy had gone unnoticed, aborted on treatment day 17. No foetal abnormalities were observed.

### Correlation between body condition scores and clinical severity scores

Body condition scores (BCS) recorded at presentation of the animals (d 0) revealed 8 horses with a score of 2, 7 horses with a score of 3, 7 horses with a score of 4 and one horse with the score of 5. With the progression of treatment and time, BCS rose and remained stable at values between 4 and 5. Clinical severity scores (CSS) recorded on day 0 were distributed as follows: Seven horses showed a score of 1, 9 horses a score of 2, 6 horses a score of 3 and 1 a score of 4. From day 60 onwards, all horses showed a CSS of 0 (Tables 1, 7 and 8).

**Table 7:** **Definition of clinical severity scores modified according to**[16]

**Severity score**	**Clinical presentation**
**0**	No pathological signs
**1**	Unilateral sero-mucous nasal discharge, palpably enlarged submandibular lymph nodes, discrete developing cutaneous nodules (n = ≤ 5) on hind quarters with tendency to ulceration on palpation, inappentence (25 % reduction in feed intake), debility, exercise intolerance (exercise tolerance test (ETT) ≤ 12 min exercise 6 min walk + 6 min trot).
**2**	Uni- or bilateral mucopurulent blood-tinged nasal discharge, nasal septum ulcers (n = ≤ 7), mild dyspnea (audible respiratory sounds from a distance of 3 m), discrete cutaneous nodules and ulcers; slightly to palpably enlarged submandibular lymph nodes, inappentence (40 % reduction in feed intake), exercise intolerance (ETT ≤ 8 min exercise; 4 min walk + 4 min trot) and cough
**3**	Bilateral blood tinged purulent nasal discharge, confluent nasal septum ulcers, moderate dyspnea (audible respiratory sound from a distance of 7 m), cutaneous nodules and ulcers in perineal region, groin, medial aspects of thighs with lymphangitis (pearly cord); visibly enlarged submandibular lymph nodes; inappentence (65% reduction in feed intake) oedema of hock joints, exercise intolerance (ETT ≤ 4 min exercise; 3 min walk +1 min trot) and cough
**4**	Bilateral copious purulent discharge with epistaxis, bilateral nasal septum ulcers with perforation, severe dyspnea (audible respiratory sounds from a distance of 10 m), cutaneous nodules and ulcers throughout the body, cording of lymphatics (pearly cords) on trunk/abdomen, legs; oedema of hind quarters and lameness, diagonal spreading in the hind quarters; inappentence (85 % reduction in feed intake), exercise intolerance (ETT ≤ 2 min exercise; 2 min walk) and cough

**Table 8:** Comparison of body condition and clinical severity scores during the study period

**d 0**				**d 60**		**d 120**		**d 180**		**d 240**		**d 300**		**d 360 - d 420**	
Score BCS*	**# of horses**	**Score CSS**	**# of horses**	**# of horses/score**											
				**BCS**	**CSS**	**BCS**	**CSS**	**BCS**	**CSS**	**BCS**	**CSS**	**BCS**	**CSS**	**BCS**	**CSS**
**1**	-	**0**	-	-	-	-	-	-	-	-	-	-	-		
**2**	8	**1**	7	-	-	-	-	-	-	-	-	-	-		
**3**	7	**2**	9	-	-	-	-	-	-	-	-	-	-		
**4**	7	**3**	6	**18**	**0**	**2**	**0**	**-**	**0**	**3**	**0**	**4**	**0**	**Maintained between 4–5 BCS**	
**5**	1	**4**	1	**3**	**0**	**13**	**0**	**8**	**0**	**11**	**0**	**12**	**0**		
**6**	-			**2**	**0**	**2**	**0**	**9**	**0**	**3**	**0**	**1**	**0**		
**7**	-			-	-	-	-	-	-	-	-	-	-	-	-
**8**	-			-	-	-	-	-	-	-	-	-	-	-	-
**9**	-			-	-	-	-	-	-	-	-	-	-	-	-

BCS: Body condition score; *for optimal performance polo horses are maintained at a level of 5; CSS: Clinical condition score.

### Induction of immunosupression

On day 90, after completion of therapy, six randomly selected horses were treated daily for 10 days with corticosteroids to induce immunosupression. During the 14 d observation period, no recrudescence of clinical signs was observed.

### Pathology and bacteriology

Necropsy revealed non specific findings, except for discrete calcified lung lesions, especially at the lower rim of the cardiac lobe and scarring of the mediastinal lymph nodes. Samples taken aseptically from the mediastinal lymph nodes, lungs tissue and from submandibular lymph nodes were negative for *B. mallei*.

### Haematological analyses

Haematological parameters were assessed in all 23 horses on day 0 (before initiation of treatment), on day 84 and during the one year follow up period (Table 9). On day 0, all horses showed a significant decrease in total RBC counts, haemoglobin levels, and PCV values, but a significantly elevated leukocyte count and an accelerated erythrocyte sedimentation rate when compared to standard hematologic values [14]. When the blood was subjected to differential leukocyte count, a significant increase in neutrophils along with a significant decrease in lymphocytes was observed, whereas eosinophils, monocytes, and basophils were within the normal range. Calculations of erythrocyte indices revealed macrocytic hypochromic anaemia (data not shown). In the course of treatment and during the follow up period red blood cell count values returned to physiological values. Differential white blood cell counts revealed an increase of neutrophils at day 360 and an increase of lymphocytes at day 180 and day 270.

**Table 9:** Hematologic values of polo horses with glanders during the study period

**Treatment period (n = 23)**			**Post treatment observation period (n = 17)**		
**Parameters**	**d 0**	**d 84**	**d 180**	**d 240**	**d 360**
**RBC count** (10^12^/1)	3.94 ± 0.78	6.30 ± 0.4	6.80 ± 0.37	7.053 ± 0.29	6.957 ± 0.36
**WBC count** (10^9^/1)	19.15 ± 4.11	8.10 ± 0.79	7.91 ± 1.23	7.97 ± 2.14	7.52 ± 1.12
**Hb [g/dL]**	7.918 ± 1.7	11.94 ± 0.69	12.79 ± 1.1	13.58 ± 1.0	13.13 ± 1.2
**PCV (ratio)**	0.25 ± 0.047	0.38 ± 0.015	0.41 ± 0.028	0.39 ± 0.025	0.38 ± 0.0 12
**ESR (mm/20 min)**	126.83 ± 10.40	25.15 ± 2.6	21.42 ± 1.94	23.19 ± 2.41	19.89 ± 3.15
**Differential Leukocyte Count**					
**Neutrophils** (%)	73.06 ± 9.12	51.0 ± 9.12	50.3 ± 4.39	53.2 ± 3.14	57.28 ± 0.89
**Lymphocytes** (%)	21.69 ± 6.95	39.7 ± 3.91	47.1 ± 3.01	43.01 ± 2.10	37.5 ± 1.23
**Eosinophils** (%)	4.77 ± 2.16	3.11 ± 0.57	2.12 ± 0.76	2.190 ± 0.911	4.23 ± 2.11
**Monocytes** (%)	1.69 ± 1.02	1.52 ± 0.89	1.76 ± 1.40	2.140 ± 1.024	1.2 ± 1.00
**Basophils** (%)	**-**	**-**	**-**	**-**	**-**

### Indirect haemagglutination assays

At day 0, prior to malleinisation, 22/23 horses showed positive titres. Titres shifted during the treatment period, reaching their peak during the first month. Titres progressively declined three months after ending of treatment. Eight months into the observation period the titres of all horses dropped to values (≤ 1:320) considered negative for glanders [17] (Table 3).

## Discussion

Glanders has been eliminated from the western world by means of a rigorous test and culling policy, frequently accompanied by sensible compensation schemes [18,19]. However, in countries with low or no compensation, albeit strict legislation, implementation of these regulations is extremely difficult, and as a consequence regions of endemicity may be formed or continue to persist [20]. These regions not only pose a local public health threat but may also have a major impact on international horse transportation, especially with regard to equestrian sport and breeding. Highly valuable animals are regularly transported to and from endemic regions and thus pose a risk of reintroduction of disease to so far disease free areas. Availability of effective treatment schemes and post-exposure prophylaxis could help to reduce spread of disease. In recent years, evidence has accumulated from *in vitro* antibiotic sensitivity testing and *in vivo* treatment studies that certain antibiotics such as enrofloxacin, erythromycin, doxycycline, and sulphonamides might be effective against *B. mallei*[1,21,22]. The present study therefore, aimed at assessing the effectiveness of a treatment protocol of 12 week duration based on the parenteral administration of enrofloxacin and trimethoprim + sulfadiazine, followed by the oral administration of doxycycline during a confined glanders outbreak in horses in Pakistan. Enrofloxacin is a fluoroquinolone antibiotic with an antibacterial activity against a broad range of Gram- positive and Gram-negative bacteria. Its mode of action is not fully known, but it is believed to interfere with bacterial DNA synthesis by inhibiting bacterial DNA gyrase [23]. Trimethoprim reversibly inhibits the bacterial synthesis of tetrahydrofolic acid, which is essential for the *de novo* synthesis of Thymidine monophosphate, a precursor of the DNA metabolite Thymidine triphosphate [23]. Sulfadiazine, like other sulphonamides, interferes with the bacterial synthesis of dihydrofolic acid, ultimately leading to bacterial death by starvation [23]. Doxycycline is a semi-synthetic tetracycline with an antibacterial activity against a broad range of Gram-positive and Gram-negative bacteria. Tetracyclines inhibit protein synthesis by binding to the 30S subunit of susceptible organisms [23].

Several rather anecdotal reports describing the successful treatment of horses with glanders exist. Early attempts towards the end of the 19^th^ century and in the very beginning of the 20^th^ century to “vaccinate” or even cure horses using mallein or other heat or chemically inactivated *B. mallei* preparations, i. e. “anamorve”, and “farase” did not lead to satisfactory results and were thus aborted [24,25]. However, a few decades later, new approaches based on the application of different sulphonamides combined with mallein or “anamorve”, i.e. heat-inactivated, and formolized *B. mallei* showed a more promising outcome. Back in 1951, Deyhimi and Katai succeeded in treating 141 glanderous horses and 18 mules by means of using various sulphonamides (sulphathiazole, sulphanilamide, sulphadiazine, sulphamezathine) for thirty consecutive days in combination with “anamorve” or mallein [26]. Subsequent post mortem studies done 12 and 30 months after end of treatment on three horses revealed non specific findings and no cultures could be obtained. Moreover, inoculation of hamsters with samples obtained from these horses did not induce any kind of reaction. A similar treatment protocol was used by Fathi et al. [27], who managed to cure 384/400 glanderous horses and mules by the combined application of sulphonamides given for 30 consecutive days with a formolized preparation of *B. mallei* or mallein. Hu et al. used sulphamezathine (sulfadimidin) and mallein to treat artificially infected acutely diseased horses and asses and apparently cured 41/48 of the former and 17/23 of the latter [28]. In a more recent study, Muhammad et al. managed to alleviate clinical symptoms in 13 draught equines (horse, mare, and mule) by applying dimethylsulfoxide, a free radical scavenger with anti-inflammatory effects and norfloxacin, a fluoroquinolone antibiotic, for four consecutive days [6]. All animals showed a temporary improvement which generally lasted for 2–3 weeks. In all studies mentioned including our own successful outcome was dependant on duration of therapy. The significance of treatment duration on disease outcome has also been shown for small laboratory animals such as mouse and hamster, and man [1,29,30]. Infected mice treated for ten days with ceftazidime, a third generation cephalosporin and levofloxacin, a fluoroquinolone, survived for 34 days, however treatment did not result in complete clearance of infection, and bacteria were still found in lungs and spleens [1]. Likewise, onset of antibiotic treatment after infection with *B. mallei* and efficacy of the antibiotic used influenced disease outcome in hamsters significantly [29]. Prolonged treatment duration possibly prevents the establishment of chronic disease. Chronic glanders as a sequel to non-apparent or non-fatal infection [24,31] poses a particular challenge to the practitioner as it can go unnoticed for a long period of time. It is still not clear what causes the “dormant state” and what triggers off disease. Histopathological studies in horses assessing samples obtained from glanderous lesions of the nasal septum have detected bacteria in the vicinity of extensive accumulations of disintegrating neutrophilic granulocytes and macrophages, indicative of intracellular invasion and survival [32]. However, recent data obtained from a respiratory cell model suggest that *B. mallei* actually induces apoptotic cell death [33]. This might offer an explanation for the protraction or recurrence of disease. Depending on the amount of released bacteria and the individual immune status of the affected animal, the disease can continue as a non-apparent infection or exacerbate.

The results of the present study suggest that long term treatment, i.e. a minimum of 12 weeks, of glanderous horses with a combination of various antibiotics could apparently eliminate the pathogenic agent from the infected animal host. Induction of immunosupression in six randomly selected horses did not lead to recrudescence of disease. Moreover, when the treated animals were returned to the polo club and housed with other horses, no further cases were reported. Cultivation is considered a method with low sensitivity, especially in chronic cases. Under ideal circumstances, one viable bacterium is hypothetically sufficient for cultivation. Based on the assumption that immunosupression will activate infection and the number of viable bacteria is high, one can assume an increase in sensitivity. In order to determine the success of treatment is not sufficient to rely on cultivation results alone; all techniques applied, i.e. the clinical picture, pathology/necropsy results, microbiology, and health status of the sentinel animals must be evaluated in their entirety.

Of the treated horses, seven are still active at the polo club and two are kept for breeding purposes and are still healthy to date (September 2012; data not shown). The remaining eight were sold and their whereabouts are unknown to the authors. A further shortcoming of this study is that no animal was submitted to immunosuppressive therapy after the one year observation treatment to induce recrudescence. However, if the horses were chronically ill, constant stress due to training and pregnancy ought to trigger off acute disease. Ever since this outbreak, horses at the polo club have been monitored regularly with the mallein test and none of the treated animals has been tested positive so far (data not shown). It must be stressed that any animal (treated or untreated) with a positive complement fixation test (CFT) or positive indirect haemagglutination test (IHAT) is subject to national and international restrictions and regulations concerning movement control, re-testing, quarantine and culling. Of note are the findings that on day 0, prior to malleinisation, 22/23 horses where positive in the IHAT, and titres increased further during the treatment period. One explanation for this rise in titres is the malleinisation, which is in accordance with previous findings [34]. A further influencing factor might be the antibiotic-induced release of *B. mallei* antigen. The gradual but continuous decline of the titres could be an indication of effective and successful therapy.

Although the presented study has shown very promising results, they must be treated with some caution. *B. mallei* strains from the Punjab represent only a small part of the genetically diverse pathogen [35]. Therefore, more studies, possibly from small confined outbreaks in different endemic regions are needed to test if this treatment regime is effective on other *B. mallei* strains.

## Conclusions

One has to bear in mind, that even if this treatment scheme should prove successful, it is expensive and therefore only an option in certain situations, e.g. valuable horses, mules, donkeys in wild life conservation scenarios. In situations in which the affected animal is the only means of income this long lasting treatment in unrealistic with regard to cost and control of medication. A more efficient means of containing spread of disease is to cull the affected animal and provide adequate compensation.

## Competing interests

The authors declare to have no competing interests.

## Authors’ contributions

Conception and design of study: MG; MS; Performance of experiments (malleinisation, susceptibility testing, bacteriology and pathology) and data collection: MS, AN, MHH, MKM, MNA, TF; Editing and revision of manuscript: HN, IK; Drafting of manuscript: MS, MG, IK; Data evaluation: MS, MG, AN, LDS; Writing of manuscript: LDS. All authors read and approved the final manuscript.

## Authors’ information

Heinrich Neubauer is the head of the OIE reference laboratory for glanders.
